# Antigen Presentation Machinery Signature-Derived CALR Mediates Migration, Polarization of Macrophages in Glioma and Predicts Immunotherapy Response

**DOI:** 10.3389/fimmu.2022.833792

**Published:** 2022-03-28

**Authors:** Rui Chen, Hao Zhang, Wantao Wu, Shuyu Li, Zeyu Wang, Ziyu Dai, Zaoqu Liu, Jian Zhang, Peng Luo, Zhiwei Xia, Quan Cheng

**Affiliations:** ^1^ Department of Neurosurgery, Affiliated Nanhua Hospital, University of South China, Hengyang, China; ^2^ Department of Neurosurgery, Xiangya Hospital, Central South University, Changsha, China; ^3^ National Clinical Research Center for Geriatric Disorders, Xiangya Hospital, Central South University, Changsha, China; ^4^ Department of Oncology, Xiangya Hospital, Central South University, Changsha, China; ^5^ Department of Thyroid and Breast Surgery, Tongji Hospital, Tongji Medical College of Huazhong University of Science and Technology, Wuhan, China; ^6^ Department of Interventional Radiology, The First Affiliated Hospital of Zhengzhou University, Zhengzhou, China; ^7^ Department of Oncology, Zhujiang Hospital, Southern Medical University, Guangzhou, China; ^8^ Department of Neurology, Hunan Aerospace Hospital, Changsa, China

**Keywords:** antigen presentation machinery, glioma, microenvironment, prognosis, genomic alteration, immunotherapy

## Abstract

Immunogenicity, influenced by tumor antigenicity and antigen presenting efficiency, critically determines the effectiveness of immune checkpoint inhibitors. The role of immunogenicity has not been fully elucidated in gliomas. In this study, a large-scale bioinformatics analysis was performed to analyze the prognostic value and predictive value of antigen presentation machinery (APM) signature in gliomas. ssGSEA algorithm was used for development of APM signature and LASSO regression analysis was used for construction of APM signature-based risk score. APM signature and risk score showed favorable performance in stratifying survival and predicting tumorigenic factors of glioma patients. APM signature and risk score were also associated with different genomic features in both training cohort TCGA and validating cohort CGGA. Furthermore, APM signature-based risk score was independently validated in three external cohorts and managed to predict immunotherapy response. A prognostic nomogram was constructed based on risk score. Risk score-derived CALR was found to mediate the invasion and polarization of macrophages based on the coculture of HMC3 and U251 cells. CALR could significantly predict immunotherapy response. In conclusion, APM signature and APM signature-based risk score could help promote the clinical management of gliomas.

## Introduction

Gliomas account for the majority of brain tumor and are one of the top-leading causes of cancer death worldwide. Although the surgical resection with adjuvant chemoradiotherapy could effectively treat gliomas to some extent, the overall survival (OS) of low grade glioma (LGG) patients is 8–10 years and the OS of glioblastoma (GBM) is about 12–14 months (1). Given that, many researchers have dedicated to exploring novel biomarkers such as isocitrate dehydrogenase (IDH) mutation, 1p19q codeletion, and O-6-methylguanine DNA methyltransferase (MGMT) promoter methylation in glioma for a better classification of glioma patients, which is more likely to fulfill precision medicine of glioma and prolong the survival of glioma patients.

Immunotherapy, represented by immune checkpoint blockage, has become a promising treatment modality in solid cancer. Compared to conventional therapy options, immune checkpoint inhibitors (ICIs) induce improved clinical responses in patients. It has been proposed that cancer cells could disguise themselves to escape immune surveillance by adopting immune checkpoint pathways. ICI critically releases the already established immune response from being suppressed by immune checkpoint and help the human immune system to recognize and eradicate tumor cells again. Several factors, namely, programmed death-ligand 1 (PD-L1) expression (2), tumor mutation burden (TMB) (3), and microsatellite instability (MSI) (4) have been confirmed to affect the ICIs response. Notably, the immunogenicity of tumor cells has been proved as a fundamental determinant of the effectiveness of ICIs, where the tumor with sufficient immunogenicity is more sensitive to ICIs. Furthermore, tumor antigenicity and antigen presenting efficiency have been identified as fundamental determinants of tumor immunogenicity (5), and antigen presentation defects were revealed to contribute to the failure of ICIs (6). Taken together, exploring the antigen processing and presenting efficiency in glioma can be significant to optimize the ICIs therapy for glioma patients.

In this study, antigen processing and presenting machinery (APM) signature genes were collected and analyzed (7). The molecular characteristics of APM signature score were explored. The prognostic value and genomic features of APM signature score and APM signature gene-based risk scores were determined. Besides, APM signature gene-based risk score showed remarkable performance in predicting the immunotherapy response of glioma patients. Our results suggested that APM signature could be a novel and effective biomarker for predicting immunotherapy response in glioma.

## Materials and Methods

### Data Collecting and Preprocessing

A total of 672 glioma samples were collected from The Cancer Genome Atlas (TCGA) and 1,013 glioma samples were collected from the Chinese Glioma Genome Atlas (CGGA), where mRNAseq_693 (693 glioma samples) and mRNAseq_325 (325 glioma samples) were combined as the CGGA dataset using the R package sva for reduction of batch effect. RNA-seq data and corresponding clinical information were downloaded from the TCGA (https://xenabrowser.net/) and the CGGA (http://www.cgga.org.cn/) datasets. LGG samples were defined as grade II and grade III gliomas, while GBM samples were defined as grade IV gliomas. Samples with incomplete overall survival information of patients were excluded. Samples with missed information of IDH status were also excluded. The detailed clinical characteristics of all the included glioma samples are provided in **Table S1**.

### Development of APM Signature Score and Risk Score

APM signature score was defined using single sample gene set enrichment analysis (ssGSEA) algorithm. A total of 28 immune infiltrating cell signatures were collected from previous study (8), and the abundance of 28 immune cells was also quantified using ssGSEA algorithm. A total of 22 immune infiltrating cell signatures were calculated based on the CIBERSORT algorithm (9). Univariate cox regression analysis was performed to determine the prognostic value of APM signature gene. LASSO regression analysis was applied to explore the feature genes for calculating risk score based on their coefficients. The different levels of APM signature score and risk score in different clinical characteristics were visualized using raincloud plot. R package pheatmap was used for construction of heatmap. Copy number variation (CNV) of APM signature score and risk score were explored using GISTIC 2.0, and the genomic events were also determined. OncoPrint was applied to depict the mutation landscape of glioma sample from TCGA using R package maftools (10).

### Immunotherapy Response

The Tumor Immune Dysfunction and Exclusion (TIDE) algorithm was applied to explore individual immunotherapy response (11), which the submap analysis was used to compare differences of risk score in anti-PD-1 response and anti-cytotoxic T lymphocyte associated antigen-4 (CTLA-4) response. The melanoma dataset (GSE78220, N = 28) and the urothelial carcinoma dataset (IMvigor 210, N = 298) were both used to calculate the risk score (12). The GBM dataset (PRJNA482620, N = 17) were also used for assessing the predictive value of risk score (13).

### Single Cell Sequencing Analysis

The detailed procedures of processing the raw data of GSE138794 were described in our previous study (14). Briefly, the data was normalized using the “NormalizeData” function and the “RunPCA” function was performed for dimension reduction. Cancer cells were identified as cells with aneuploid using the R package “copykat” (15). The R package “Single R” was used to identify the immune and stromal cell types. Vlnplot, Dimplot, and Featureplot were used for visualization.

### Western Blotting Assay

The expression level of CALR and β-actin were assessed by the western blotting assay. Anti-CALR (Rabbit, 1:1,000, Proteintech, China) and anti-β-actin (Mouse, 1:5,000, Proteintech, China) were used as the primary antibody. HRP goat anti-mouse IgG (Mouse, 1:5,000, Proteintech, China) and HRP goat anti-rabbit IgG (Rabbit, 1:6,000, Proteintech, China) were used as the secondary antibody. ECL development was used for visualization.

### RT-qPCR Assay

The primers of β-actin (F ACCCTGAAGTACCCCATCGAG; R AGCACAGCCTGGATAGCAAC) and CALR (F GCCGCGCCAAATAATGTCTC; R ATCCACCCCAAATCCGAACC) were designed using the primer 5.0. Total RNAs were extracted and reversely transcribed into cDNA by HiScript Q RT SuperMix for RT-qPCR. The expression levels of β-actin and CALR were quantified using 2^−ΔΔCT^.

### Coculture of HMC3 and U251 Cells for Transwell Assay

U251 cells were digested and resuspended using 10% DMEM, and were added to the lower chamber. At the density of 1 × 10^5^ each well, U251 cells were transfected with si-NC and si-CALR. After U251 cells were transfected for 48 h, and HMC3 cells were also digested and resuspended at the density of 2 × 10^5^ each well. At the ratio of 1:1, U251 cells and HMC3 cells were cocultured for 48 h. After being washed with phosphate buffer saline (PBS) for twice, the upper chamber was fixed using acetone and methyl alcohol at the ratio of 1:1 for 20 min. The upper chamber was then stained with 0.5% crystal violet for 5 min for photographing.

### Coculture of HMC3 and U251 Cells for Immunofluorescence Staining

The transfected U251 cells on the upper chamber were cocultured with HMC3 cells on the cell slide of the lower chamber at the ratio of 1:1. After being washed with phosphate buffer saline (PBS), one group of cell slides were incubated with primary antibody CD68, CD11c and the other group of cell slides were incubated with primary antibody CD68, CD163. The cell slides were subsequently incubated with anti-mouse and anti-rabbit IgG secondary antibody. The cell nucleus of cocultured cells was stained with DAPI. The cell slides were observed with microscope and representative images were photographed.

### Statistical Analysis

All statistical analyses were performed on R project. Group comparisons were determined by two‐tailed t-test or one‐way ANOVA. Spearman correlation analysis was applied to determine significant correlation between linear variables. The R package survival ROC was applied to plot the ROC curves (16). Survival analysis was visualized using the Kaplan–Meier curves. Univariate cox regression analysis was performed using the R package survival. All tests with P-values <0.05 were considered to be statistically significant.

## Results

### Molecular Features and Clinical Characteristics of APM Signature Score

**Figure 1:**
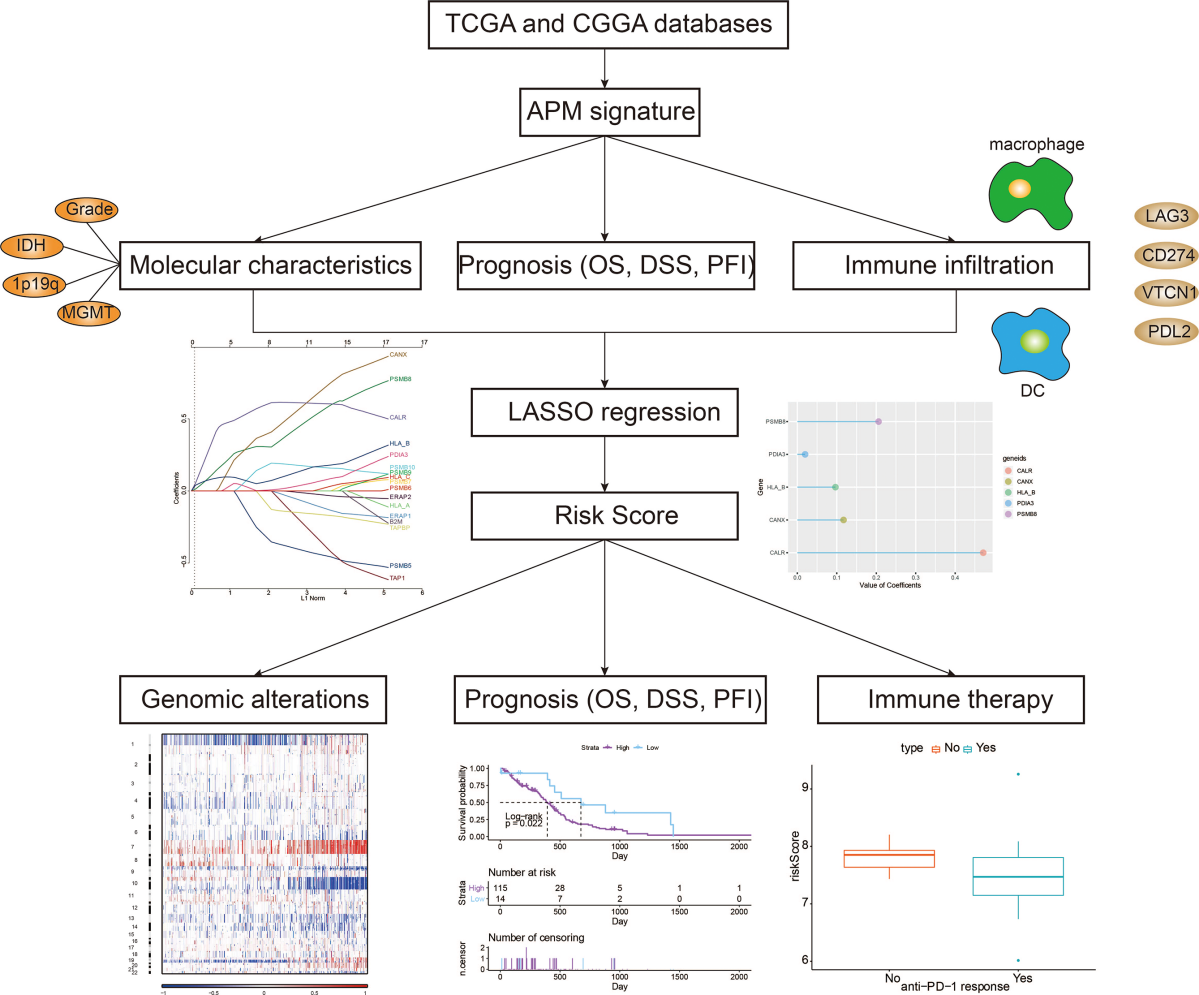
Flow diagram of this study.

**Figure 2:**
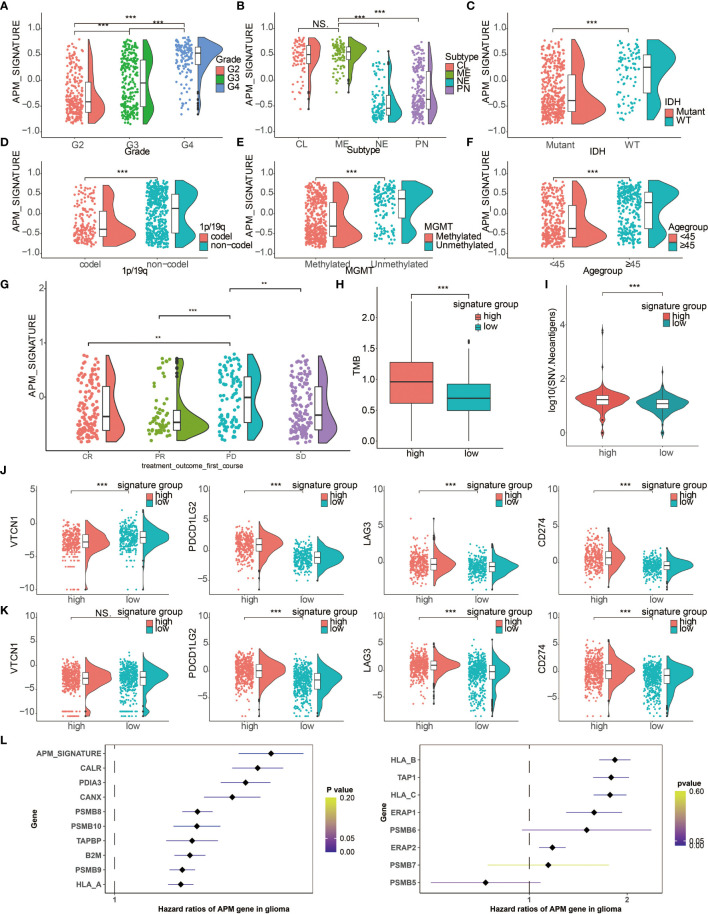
Clinical characteristics and molecular features of APM signature. Raincloud plot depicting the expression differences of APM signature in **(A)** tumor grade, **(B)** molecular subtypes, **(C)** IDH status, **(D)** 1p19q status, **(E)** MGMT status, **(F)** age groups, and **(G)** treatment outcome. CR, Complete Remission/Response; PR, Partial Remission/Response; PD, Progressive Disease; SD, Stable Disease. **(H)** Different levels of TMB in two APM signature groups. **(I)** Different levels of SNV neoantigens in two APM signature groups. **(J)** Expression differences of VTCN1, PDCD1LG2, LAG3, and CD274 in two APM signature groups in the TCGA. **(K)** Expression differences of VTCN1, PDCD1LG2, LAG3, and CD274 in two APM signature groups in the CGGA. **(L)** Forest plot depicting the hazard ratios of APM genes and APM signature score in glioma samples. NS, Not Statistically Significant; **P < 0.01; ***P < 0.001.

The overall study design is shown in **Figure 1**. Based on a previous review paper about APM (17), the following genes were collected for estimation of APM signature: PSMB5, PSMB6, PSMB7, PSMB8, PSMB9, PSMB10, TAP1, TAP2, ERAP1, ERAP2, CANX, CALR, PDIA3, TAPBP, B2M, HLA-A, HLA-B and HLA-C. These signature genes were responsible for four main tasks of MHC class I antigen processing and presentation, namely, peptide generation and trimming (PSMB5, PSMB6, PSMB7, PSMB8, PSMB9, PSMB10, ERAP1, ERAP2), peptide transport (TAP1, TAP2), assembly of the MHC class I loading complex (CANX, CALR, PDIA3, TAPBP), and antigen presentation (B2M, HLA-A, HLA-B, HLA-C). The APM signature score of each glioma patient from TCGA was calculated. The increasing tumor grade of gliomas was associated with increasing APM signature score (**Figure 2A**). The molecular subtypes of GBM can be classified into classical (CL), mesenchymal (ME), neural (NE), proneural (PN). Notably, malignant subtypes, CL and ME, were associated with higher levels of APM signature score in pan-glioma, LGG, and GBM samples, respectively (**Figure 2B** and **Figure S3A**). IDH wildtype, an indicator of worse survival, was also associated with higher APM signature score in pan-glioma, LGG, and GBM samples, respectively (**Figure 2C** and **Figure S3B**). Consistently, 1p19q non-codeletion, an indicator of worse survival, was associated with higher APM signature score in pan-glioma and LGG samples, respectively (**Figure 2D** and **Figure S3C**). Besides, unmethylated MGMT promoter correlated with higher APM signature score in pan-glioma and LGG samples, respectively (**Figure 2E** and **Figure S3D**). Glioma patients aged over 45 years old had higher APM signature score in pan-glioma, LGG, and GBM samples, respectively (**Figure 2F**). Although the statistical analysis was not significant in GBM due to the small sample size, the tendency was still obvious. Taken together, the close association between APM signature and clinicopathological features was not reflected by tumor heterogeneity between LGG and GBM. So, APM signature could be successfully applied to pan-glioma samples. Additionally, glioma patients with progressive disease after treatment had higher APM signature score (**Figure 2G**).

TMB was also associated with higher level of higher APM signature score (**Figure 2H**), which suggested that APM signature score could predict ICI response in gliomas. Besides, SNV neoantigens were associated with higher level of higher APM signature score (**Figure 2I**), indicating that APM signature score could predict tumor antigenicity. Moreover, classical immune checkpoint molecules, namely, VTCN1, PDCD1LG2, LAG3, and CD274, were all highly associated with higher level of APM signature score in the TCGA and the CGGA (**Figures 2J, K**, respectively).

### Prognostic Value of APM Signature Score

**Figure 3:**
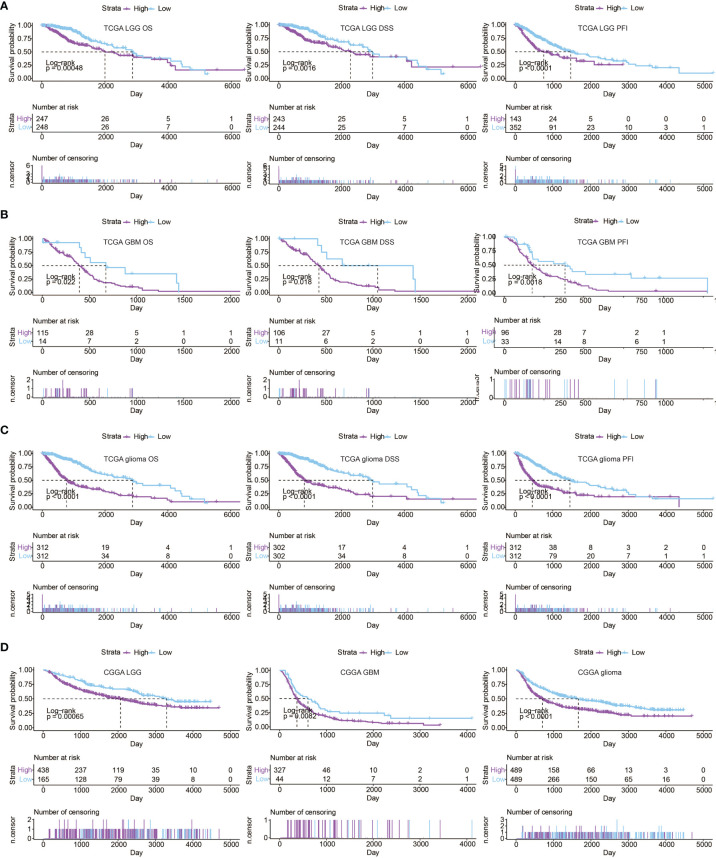
Prognostic value of APM signature score in **(A)** LGG samples from the TCGA, **(B)** GBM samples from the TCGA, **(C)** glioma samples from the TCGA. **(D)** Kaplan–Meier curves of the two APM signature score groups regarding OS of glioma samples from the CGGA.

To further determine the prognostic value of APM signature score, univariate cox regression analysis was performed on the APM signature genes. Notably, all APM signature genes except PSMB5, PSMB6, and PSMB7 were significant hazardous markers in the TCGA (**Figure 2L**). LGG patients, GBM patients, and glioma patients with high APM signature scores experienced reduced OS, disease specific survival (DSS), and progression free interval (PFI) in the TCGA (**Figures 3A–C**, respectively). Consistently, LGG patients, GBM patients, and glioma patients with high APM signatures scores experienced reduced OS in the CGGA (**Figure 3D**). The expression differences of APM signature genes in different clinical factors of glioma patients from the TCGA and the CGGA are shown in **Figures S1A, B**. Specifically, APM signature genes were more expressed in glioma patients with IDH wildtype in the TCGA (**Figure S2A**) and the CGGA (**Figure S2B**). APM signature genes were abundantly expressed in GBM patients compared to LGG patients in the TCGA (**Figure S2C**) and the CGGA (**Figure S2D**). Furthermore, APM signature genes were abundantly expressed in grade 3 glioma patients compared to grade 2 glioma patients in the TCGA (**Figure S2E**) and the CGGA (**Figure S2F**).

### Immune Infiltration Characteristics of APM Signature Score

Given the vital role of tumor immune microenvironment, the association between APM signature score and immune infiltration was explored. The expression patterns of immune infiltration cells in different levels of APM signature is shown in **Figure S1C**, which immune suppressive cells such as neutrophils, macrophages, mast cells, and regulatory T cells (Tregs) were all abundantly existed in glioma patients with high APM signature scores. DC and macrophages ranked top 2 among the immune infiltration cells positively correlated with APM signature score (**Figure S1D**). The expression patterns of inflammatory signature genes in different levels of APM signature score is shown in **Figure S1E**. APM signature score was found to be positively associated with inflammatory activities regulated by MHC I, MHC II, STAT1, interferon, LCK, HCK, and negatively associated with IgG (**Figure S1F**).

### Construction of APM Signature Gene-Based Risk Score

**Figure 4:**
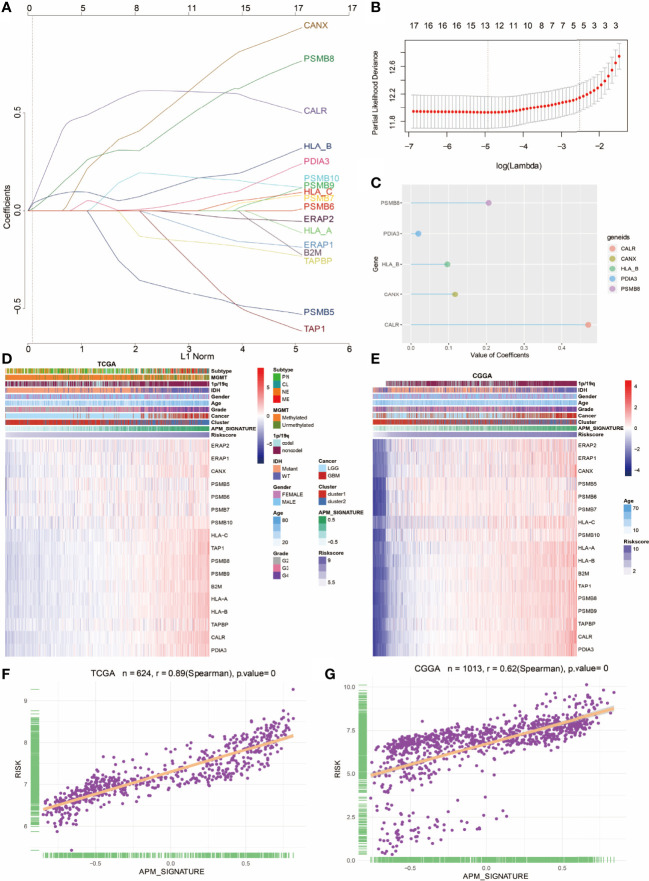
Construction of APM signature gene-based risk score. **(A)** LASSO regression analysis showing the coefficients of APM signature genes. **(B)** Partial likelihood deviance of APM signature genes. **(C)** Bubble plot depicting the APM-derived risk signature genes. **(D)** Heatmap depicting the association between APM signature genes and risk score in the TCGA. **(E)** Heatmap depicting the association between APM signature genes and risk score in the CGGA. **(F)** The correlation between APM signature and risk score in TCGA. **(G)** The correlation between APM signature and risk score in the CGGA.

LASSO regression analysis was further performed on the 17 APM signature genes (**Figure 4A**). The formula of the risk score was as follows, risk score = 0.4712 ∗ CALR (gene expression level) + 0.1171*CANX + 0.2059 ∗ PSMB8 + 0.0198 ∗ PDIA3 + 0.0966 ∗ HLA_B. The partial likelihood deviance of APM signature genes is shown in **Figure 4B**. Bubble plot was used to show the value of coefficients of 5 identified prognostic genes (**Figure 4C**). The risk score of each glioma patient from the TCGA and the CGGA was calculated on the basis of the 5 prognostic genes. Heatmap showed the consistency among APM signature gene expression values, APM signature, and risk score in the TCGA and the CGGA (**Figures 4D, E**, respectively). As expected, risk score highly correlated with APM signature score in the TCGA and the CGGA (**Figures 4F, G**, respectively).

### Molecular Features and Clinical Characteristics of Risk Score

To comprehensively determine the prognostic value of APM signature gene-based risk score, the association between risk score and different clinicopathologic factors was analyzed. The increasing tumor grade of gliomas was associated with increasing risk score (**Figure S4A**). Notably, CL and ME subtypes were associated with higher levels of risk score (**Figure S4B**). IDH wildtype was also associated with higher risk score (**Figure S4C**). Consistently, 1p19q non-codeletion was associated with higher risk score (**Figure S4D**). Besides, unmethylated MGMT promoter correlated with higher risk score (**Figure S4E**). Glioma patients aged more than 45 years old had higher risk score (**Figure S4F**). Additionally, glioma patients with progressive disease after treatment had higher risk score (**Figure S4G**).

TMB was also associated with higher level of higher risk score (**Figure S4H**), which suggested that risk score could predict ICI response in gliomas. Besides, SNV neoantigens were associated with higher level of higher risk score (**Figure S4I**), indicating that APM signature score could predict tumor antigenicity. Moreover, classical immune checkpoint molecules, namely, VTCN1, PDCD1LG2, LAG3, and CD274, were all highly associated with higher level of risk score in the TCGA and the CGGA (**Figures S4J, K**, respectively).

### Prognostic Value of Risk Score

**Figure 5:**
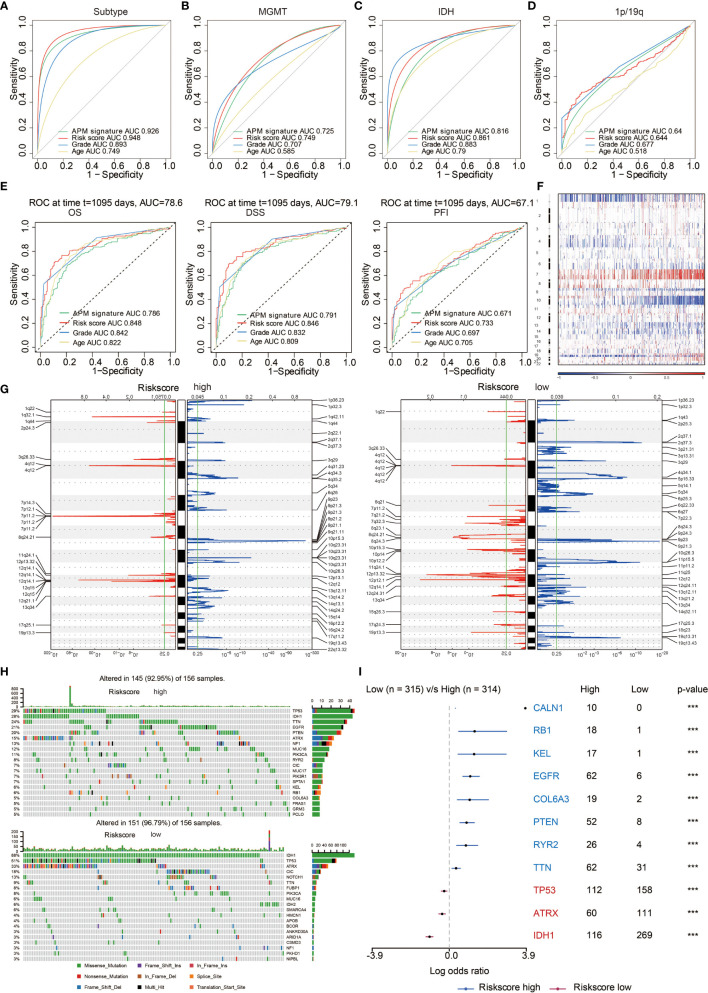
Genomic features of risk score groups. **(A)** ROC curve depicting the predictive value of APM signature and risk score in molecular subtypes. **(B)** ROC curve depicting the predictive value of APM signature and risk score in MGMT status. **(C)** ROC curve depicting the predictive value of APM signature and risk score in IDH status. **(D)** ROC curve depicting the predictive value of APM signature and risk score in 1p19q status. **(E)** ROC curve depicting the predictive value of APM signature and risk score in OS, DSS, and PFI. **(F)** The overall somatic alteration pattern of glioma. **(G)** Copy number variations in high and low risk score group. **(H)** Genomic alterations in high and low risk score group. **(I)** Forest plot depicting the differences between high and low risk score group regarding altered genes based on chi-square test. ***P < 0.001.

The ROC curves showed that risk score and APM signature score could sensitively predict molecular subtypes (**Figure 5A**), MGMT promoter methylation status (**Figure 5B**), IDH status (**Figure 5C**), and 1p19q status (**Figure 5D**). Besides, APM signature score could sensitively predict OS, DSS, and PFI with high AUC value of 0.786, 0.791, and 0.671, respectively, while risk score could sensitively predict OS, DSS, and PFI with high AUC value of 0.848, 0.846, and 0.733, respectively (**Figure 5E**). LGG patients, GBM patients, and glioma patients with high risk scores experienced reduced OS, DSS, and PFI in the TCGA (**Figures S5A–C**, respectively). Consistently, LGG patients, GBM patients, and glioma patients with high risk scores experienced reduced OS in the CGGA (**Figure S5D**).

### Genomic Features of APM Signature Score and Risk Score

Gene mutation has always been the critical factor in facilitating tumor growth, tumor progression, and tumor metastasis. The overall somatic mutation profile of glioma patients in the increase of APM signature score is shown in **Figure S6A**. The copy number variation (CNV) regions in high and low APM signature score groups are shown in **Figure S6B**, where amplifications in 1q32.1, 4q12, and 7p11.2 more frequently occurred in high APM signature score group while deletions in 2q37.3, 11p15.5, and 19q13.31 more frequently occurred in low APM signature score group. Specifically, mutations in oncogenes, EGFR (24%) and TTN (22%), more frequently occurred in high APM signature score group. On the contrary, mutations in tumor suppressors, IDH1 (89%) and TP53 (54%), more frequently occurred in low APM signature score group (**Figure S6C**).

Likewise, the genomic features of risk score were explored. The overall somatic mutation profile of glioma patients in the increase of risk score is shown in **Figure 5F**. The copy number variation (CNV) regions in high and low risk score groups are shown in **Figure 5G**, where amplifications in 1q32.1, 4q12, 7p11.2 and deletion in 9p21.3 more frequently occurred in high risk score group while amplification in 12q14.1 and deletions in 2q37.3, 11p15.5, 19q13.31 more frequently occurred in low risk score group. Specifically, mutations in oncogenes, EGFR (21%) and TTN (24%), more frequently occurred in high risk score group. On the contrary, mutations in tumor suppressors, IDH1 (88%) and TP53 (51%), more frequently occurred in low risk score group (**Figure 5H**). The differentially altered genes in the two risk score groups with statistical significance are exhibited in **Figure 5I**.

### Risk Score Could Predict Anti-PD-1 Immunotherapy Response

**Figure 6:**
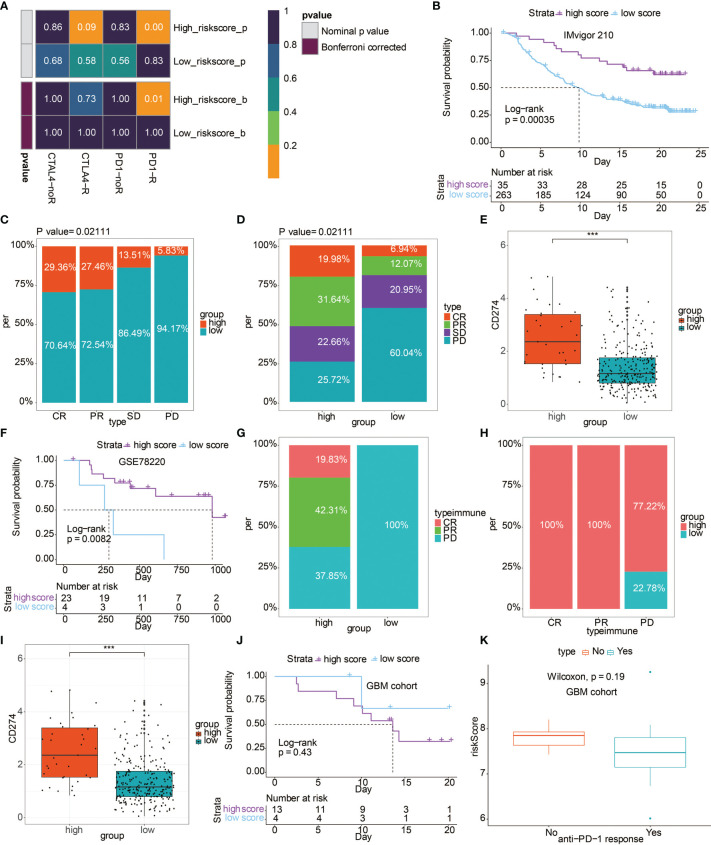
The predictive value of risk score in immunotherapy. **(A)** Submap analysis showed a significant difference in CTLA-4 and anti-PD-1 therapy response based on the TIDE algorithm regarding the risk score. **(B)** Kaplan–Meier curves of the two risk score groups regarding OS in IMvigor 210 cohort. **(C)** Box plot showing the occupation of two risk score groups in different anti-PD-1 immunotherapy responses in IMvigor 210 cohort. **(D)** Box plot showing the occupation of different anti-PD-1 immunotherapy responses in two risk score groups in IMvigor 210 cohort. **(E)** Box plot showing the expression differences of CD274 in two risk score groups in IMvigor 210 cohort. **(F)** Kaplan–Meier curves of the two risk score groups regarding OS in GSE78220 cohort. **(G)** Box plot showing the occupation of different anti-PD-1 immunotherapy responses in two risk score groups in GSE78220 cohort. **(H)** Box plot showing the occupation of two risk score groups in different anti-PD-1 immunotherapy responses in GSE78220 cohort. **(I)** Box plot showing the expression differences of CD274 in two risk score groups in GSE78220 cohort. **(J)** Kaplan-Meier curves of the two risk score groups regarding OS in GBM cohort. **(K)** Box plot showing the different levels of risk score in two anti-PD-1 response groups in GBM cohort. ***P < 0.001.

Based on the TIDE algorithm, high risk score group showed significantly better anti-PD-1 response (**Figure 6A**). In IMvigor 210 cohort, patients with high risk score experienced prolonged OS (**Figure 6B**). Patients with complete response or partial response to immunotherapy had higher occupation of risk score (**Figure 6C**). Correspondingly, patients in high risk score group were more likely to respond to immunotherapy (**Figure 6D**). Besides, the high risk score group had higher level of CD274 (**Figure 6E**). Additionally, in the GSE78220 cohort, patients with high risk score experienced prolonged OS (**Figure 6F**). The high risk score group had more patients with complete response or partial response to immunotherapy (**Figure 6G**). Correspondingly, patients with response to immunotherapy had higher occupation of risk score (**Figure 6H**). Likewise, the high risk score group had a higher level of CD274 (**Figure 6I**). Notably, in the GBM cohort receiving anti-PD-1 therapy, although the difference is not statistically significant, patients with high risk score experienced reduced OS compared to patients with low risk score (**Figure 6J**). GBM patients with no response to immunotherapy had relatively higher risk score (**Figure 6K**).

### Construction of a Nomogram Based on Risk Score

**Figure 7:**
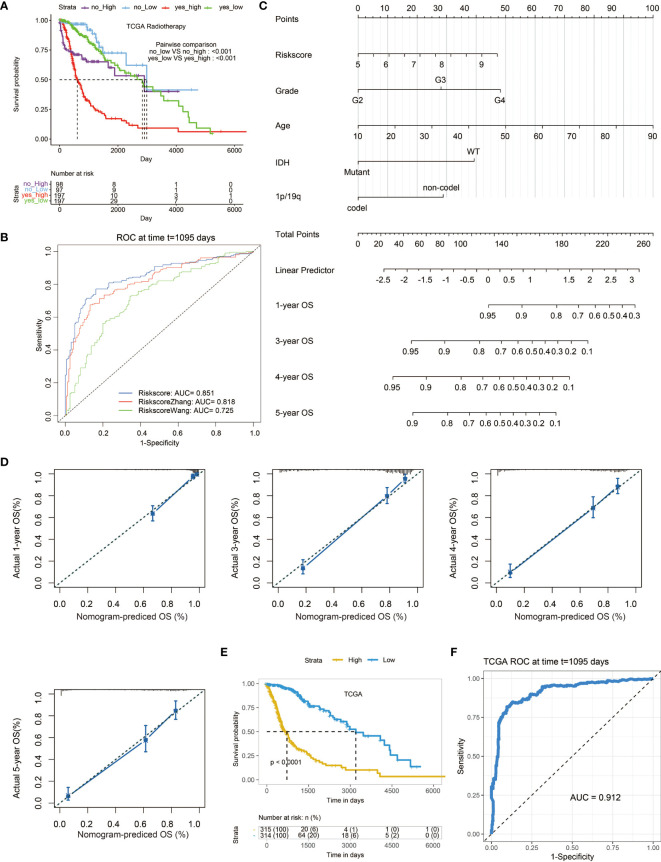
The prognostic value of risk score. **(A)** Kaplan–Meier curves of the two risk score groups receiving radiotherapy or not regarding OS in TCGA. **(B)** ROC curves depicting the predictive value of risk score compared with two previously developed signatures for 3-year survival. **(C)** A nomogram based on several clinical factors. **(D)** 1-, 3-, 4-, and 5-year calibration curves for the nomogram. **(E)** Kaplan–Meier curves of the two nomogram-based score groups regarding OS in the TCGA. **(F)** ROC curve depicting the predictive value of the nomogram-based score for 3-year survival.

To further determine the value of risk score in clinical application, we compared the survival between patients with or without radiotherapy in two risk score groups separately. It turned out that patients receiving radiotherapy with high risk score had the worst survival outcome (**Figure 7A**). We also compared our risk score with two previously established models (**Figure 7B**) (18, 19). Notably, our risk score showed the highest AUC value of 0.851 in predicting survival outcome compared with those of the two models (AUC = 0.818,0.725, respectively). Subsequently, univariate cox regression analysis was performed, which risk score was an independent clinical factor as tumor grade, age, IDH status, 1p19q status (**Table S2**). Given that, a prognostic nomogram based on these independent clinical factors was constructed (**Figure 7C**). The 1-, 3-, 4-, and 5-year calibration curves showed the reliability of the nomogram (**Figure 7D**). As expected, patients with high score based on the nomogram were associated with decreased survival (**Figure 7E**). Moreover, the nomogram-based score showed remarkable predictive value in survival outcome with the AUC value of 0.912 (**Figure 7F**).

### The Predictive Value of CALR in Immunotherapy Response

**Figure 8:**
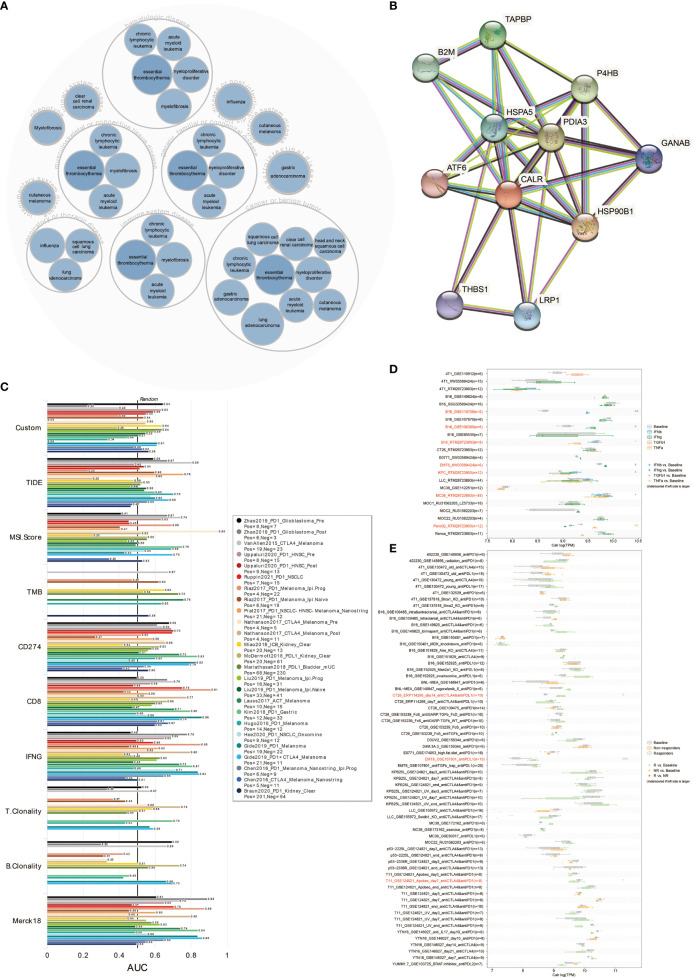
CALR predicts immunotherapy response. **(A)** Disease network related to CALR. **(B)** Protein–protein interaction network related to CALR. **(C)** The predictive value of CALR in human immunotherapy cohorts. **(D)** The predictive value of CALR in cytokine treatment. **(E)** The predictive value of CALR in murine immunotherapy cohorts.

The disease network related to CALR was generated using the Open Targets Platform (https://platform.opentargets.org), which CALR was involved in the carcinogenesis of various cancers (**Figure 8A**). The protein interaction of CALR was visualized using the STRING database (https://string-db.org/cgi/input.pl) (**Figure 8B**).

The predictive value of CALR expression in immunotherapy response was first explored in human immunotherapy cohorts. The results showed that CALR alone had an AUC of >0.5 in 17 of the 25 immunotherapy cohorts (**Figure 8C**). Moreover, CALR showed a higher predictive value than TMB, T. Clonality and B. Clonality, which gave AUC >0.5 in eight, nine and seven immunotherapy cohorts, respectively. However, CALR had lower predictive value scores than the CD274 score (AUC >0.5 in 21 immunotherapy cohorts), CD8 score (AUC >0.5 in 21 immunotherapy cohorts), and Merck 18 score (AUC >0.5 in 18 immunotherapy cohorts).

To investigate the correlation between CALR expression in tumor cells and their sensitivity to immunotherapy agents, CALR expression level across tumor cell-lines were compared between pre- and post-cytokine treated samples. As shown in **Figure 8D**, IFNγ treatment significantly upregulated CALR expression in B16 and EMT6 cells, IFNβ treatment significantly upregulated CALR expression in KPC while downregulated CALR expression in MC38 cells, TNFα treatment significantly upregulated CALR expression in Panc-02 while downregulated CALR expression in B16 cells. We then compared the CALR expression level across different tumor cell-lines between pre- and post-ICB treatment and responders and non-responders. CALR expression was significantly increased in CT26 cells that responded to anti-CTLA and anti-PD-1 treatment, and CALR expression was obviously increased in EMT6 cells that responded to anti-PD-1 treatment. On the contrary, CALR expression was significantly decreased in T11 cells that responded to anti-PD-1 treatment (**Figure 8E**). Above all, CALR expression can function as an effective biomarker for the prediction of immunotherapy response.

### Validation of CALR in Mediating the Invasion of Macrophages

**Figure 9:**
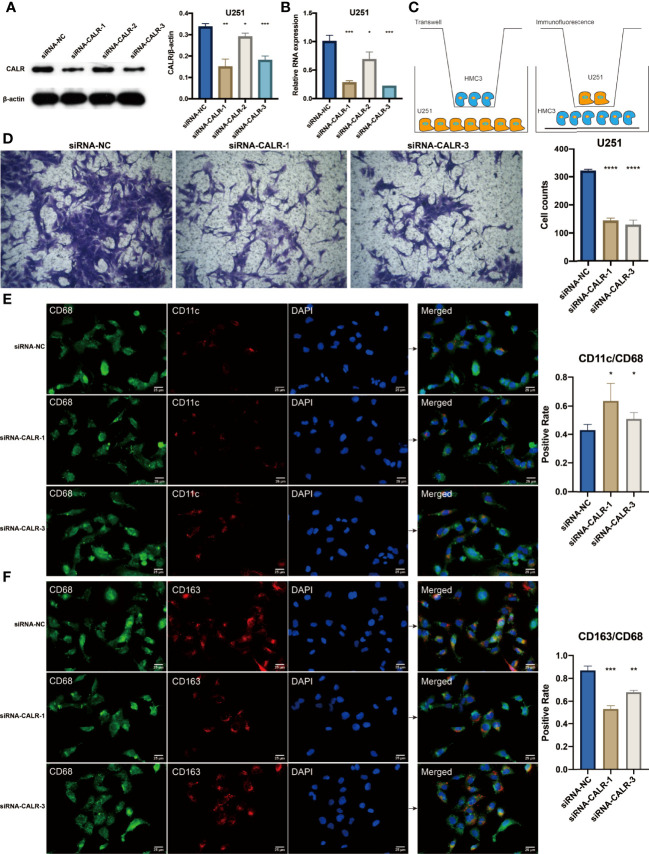
**(A)** Western blotting results for U251 cells treated with siRNAs. Statistical analysis of the western blotting results in different siRNA groups. **(B)** Statistical analysis of the qPCR results in different siRNA groups. **(C)** Diagram for the coculture between HMC3 and U251 cells. **(D)** Transwell assay for the cocultured HMC3 cells. Statistical analysis of the migrated HMC3 cells in different siRNA groups. **(E)** Immunofluorescence staining of CD68 and CD11c in HMC3 cells. **(F)** Immunofluorescence staining of CD68 and CD163 in HMC3 cells. *P < 0.05; **P < 0.01; ***P < 0.001; ****P < 0.0001.

As the major component of the LASSO regression-based genes, CALR had the highest coefficient in determining the risk score. Therefore, CALR was hypothesized to play a vital role in the tumor microenvironment of glioma. In the TCGA, glioma patients with high CALR expression were associated with decreased survival (**Figure S7A**). As mentioned above, macrophage was one of the top two immune cells exhibiting significant correlation with APM signature score. We next tried to establish the connection between CALR and macrophage. In the TCGA, macrophages were more active in glioma patients with high CALR expression (**Figure S7B**). Besides, in the GBM single cell sequencing dataset GSE138794 (**Figure S7C**), CALR was abundantly expressed in cancer cells and macrophages based on Dimplot and Vlnplot (**Figures S7D, E**). Based on the western blotting assay in U251 cell line, three siRNA targeting CALR significantly suppressed the protein expression of CALR (**Figure 9A**). qPCR assay further validated the results (**Figure 9B**). siRNA-1 and siRNA-3 were the top two interference efficient siRNA in suppressing CALR, and they were used for subsequent experiment. The diagram for coculture between HMC3 and U251 cells is shown in **Figure 9C**. After coculturing HMC3 and U251 cells, macrophages in siRNA-1 and siRNA-3 groups showed significantly decreased ability in invasion compared with siRNA-NC group (**Figure 9D**). Moreover, macrophages in siRNA-1 and siRNA-3 groups were more likely to polarize into M1 type macrophages (**Figure 9E**). In the meanwhile, macrophages in siRNA-1 and siRNA-3 groups were less likely to polarize into M2 type macrophages (**Figure 9F**).

## Discussion

Immunogenicity, influenced by both tumor cell itself and the surrounding tumor microenvironment, has been reported to be critical inherent feature of cancer and closely connected to immunotherapy response. Tumor antigenicity and antigen presenting ability are two key determinants of tumor immunogenicity. In this study, we employed APM signature genes from previous study to measure the immunogenicity of gliomas. APM signature score and the corresponding risk score showed favorable performance in stratifying survival and predicting tumorigenic factors of glioma patients. APM signature score and risk score were also associated with different genomic features. Furthermore, APM signature-based risk score managed to predict immunotherapy response.

The APM signature score was developed based on the expression value of CALR, PDIA3, CANX, PSMB8, PSMB10, TAPBP, B2M, PSMB9, HLA-A, HLA-B, TAP1, HLA-C, ERAP1, PSMB6, ERAP2, PSMB7, and PSMB5. PDIA3 and B2M have been identified as critical immune modulators and hazardous markers in gliomas (20, 21). CANX has also been identified as prognostic marker in LGG (19). Proteasome beta subunits (PSMB) family is identified as a negative regulator of innate immune responses (22). HLA families are important mediators in cancer immunity (23). CALR mutation was frequently detected in different cancer types (24). The endoplasmic reticulum (ER) aminopeptidases ERAP1 and ERAP2 are two frequently altered genes that affect anti-tumor immune responses and tumor growth (25). Moreover, the downregulation of TAP1 has been reported to elicit immune escape in colorectal cancer (26).

As immunogenicity is an important inherent feature of tumor cells, the constructed APM signature score and risk score were found to be more associated with higher tumor grade, CL and ME subtypes, IDH wildtype, 1p19q non-codeletion, unmethylated MGMT promoter as expected, all of which indicated more malignancy of gliomas. It should be noted that high APM signature score and high risk score were both associated with more copy number variations, including 1q32.1 (NR5A2), 4q12 (KIT), and 7p11.2 (EGFR). Besides, tumor suppressors, including IDH1 and TP53, were more frequently occurred in low APM signature score group and low risk score group. Moreover, APM signature score and risk score predicted worse survival outcome of glioma patients. Taken together, these results suggested that APM signature score and risk score could be reliable markers in predicting malignancy of gliomas and prognosis of glioma patients.

Tumor immune microenvironment plays an important role in regulating the tumorigenic process and immunogenic process of tumor (27, 28). As expected, APM signature score correlated with multiple immune suppressive cells, namely, neutrophils, Tregs, mast cells, and macrophages. It should be noted that patients with higher grade of gliomas were estimated with relatively higher level of immune infiltration cells, namely, T cells, B cells, DC, and macrophages (29, 30), all of which have been proved with robust antigen presentation capacity and could express antigen presentation machinery. This was in accordance with our finding that higher grade of gliomas was associated with higher level of APM signature. APM signature score also correlated with inflammatory activities by regulating the T cell mediated antigen presenting process. Additionally, immune checkpoint molecules have already been proved to help facilitate the immune escape of tumor cells (31). The strong positive correlation between APM signature-based risk score and classical immune checkpoint molecules including VTCN1, PDCDLG2, LAG3, and CD274 further indicated that APM signature score could predict an immunosuppressive and onco-inflammatory microenvironment that supported tumor growth and progression.

Given the vital role of APM signature score and risk score in immunity, we explored the predictive value of risk score in immunotherapy response. Previous study has proved that APM signature score could predict response to immune checkpoint blockage in non-small cell lung cancer (NSCLC) and melanoma (32). Based on the TIDE algorithm, glioma patients with high risk score were more likely to respond to anti-PD-1 immunotherapy. In two most widely studied cohorts receiving anti-PD-1 therapy, IMvigor 210 and GSE78220, high risk score predicted better survival outcomes, better immunotherapy responses, and higher expression levels of CD274. These results confirmed the remarkable predictive value of risk score in anti-PD-1 response. Although immunotherapy has demonstrated promising results in several solid cancer types, the clinical outcome of GBM patients receiving immunotherapy is still dismal. Based on a recent GBM cohort receiving anti-PD-1 therapy, high risk score correlated with insignificant worse survival and worse immunotherapy responses. The insignificant differences between high risk score group and low risk score group can be attributed to the insufficient sample size. The contradictory role of risk score in GBM compared to those in melanoma and urothelial carcinoma may partly be explained by the different tumor microenvironment in central nervous system. Therefore, more GBM cohorts with large-scale samples are urgently needed for exploring the clinical practice of immunotherapy.

By comparing our risk score with other previous signatures, our risk score demonstrated its advantage in predicting survival outcome of patients. A prognostic nomogram was also constructed to further prove the clinical value of risk score. In addition, as the core component of risk score, CALR was proved to mediate the invasion and polarization of macrophages in gliomas. CALR could also effectively predict immunotherapy response in both human and murine immunotherapy cohorts. Specifically, CALR expression was significantly increased in CT26 cells (murine colorectal cancer) that responded to anti-CTLA and anti-PD-1 treatment, and CALR expression was obviously increased in EMT6 cells (murine breast cancer) that responded to anti-PD-1 treatment. On the contrary, CALR expression was significantly decreased in T11 cells (genetically engineered mouse models (GEMM) of mammary cancer with overexpression of murine APOBEC3) that responded to anti-PD-1 treatment (33). Thus, CALR was proposed to be affected by APOBEC3B.

In conclusion, our study demonstrated that APM signature score and APM signature-based risk score could be potential markers in predicting survival outcome, IDH status, 1p19q status, MGMT status, and molecular subtypes of glioma patients. Besides, APM signature score could be associated with an immune suppressive microenvironment and risk score could potentially predict immunotherapy responses of patients. However, the role of APM signature in immunotherapy of GBM needs to be further elucidated. It is expected that APM signature score and APM signature-based risk score could help promote the clinical management of gliomas.

## Data Availability Statement

The datasets presented in this study can be found in online repositories. The names of the repository/repositories and accession number(s) can be found in the article/**Supplementary Material**.

## Author Contributions

Conception and design: HZ, ZX, QC and RC. Foundation support: QC, ZX and RC. Experiment: HZ, WW and SL. Acquisition and analysis of data: HZ, WW, ZD, ZW and RC. Interpretation of data: HZ, RC. Drafting the manuscript and revising for submission quality: HZ, RC, ZL, JZ and PL. Reviewing and approving the final vision: All authors. Study supervision: QC, RC.

## Funding

This work was supported by the Hunan Provincial Natural Science Foundation of China (Nos. 2019JJ80056), the Science Foundation of AMHT Group (No. 2020YK10).

## Conflict of Interest

The authors declare that the research was conducted in the absence of any commercial or financial relationships that could be construed as a potential conflict of interest.

## Publisher’s Note

All claims expressed in this article are solely those of the authors and do not necessarily represent those of their affiliated organizations, or those of the publisher, the editors and the reviewers. Any product that may be evaluated in this article, or claim that may be made by its manufacturer, is not guaranteed or endorsed by the publisher.

## Abbreviations

OS, overall survival; LGG, low grade glioma; GBM, glioblastoma; IDH, isocitrate dehydrogenase; MGMT, O-6-methylguanine DNA methyltransferase; ICIs, immune checkpoint inhibitors; PD-L1, programmed death-ligand 1; TMB, tumor mutation burden; MSI, microsatellite instability; APM, antigen processing and presenting machinery; TCGA, The Cancer Genome Atlas; CGGA, Chinese Glioma Genome Atlas; ssGSEA, single sample gene set enrichment analysis; CNV, copy number variation; TIDE, Tumor Immune Dysfunction and Exclusion; CTLA-4, anti-cytotoxic T lymphocyte associated antigen-4; CL, classical; ME, mesenchymal; NE, neural; PN, proneural; Tregs, regulatory T cells; DSS, disease specific survival; PFI, progression free interval; ER, endoplasmic reticulum; PSMB, proteasome beta subunits; NSCLC, non-small cell lung cancer.
