# Corrigendum: Antigen Presentation Machinery Signature-Derived CALR Mediates Migration, Polarization of Macrophages in Glioma and Predicts Immunotherapy Response

**DOI:** 10.3389/fimmu.2022.931433

**Published:** 2022-06-02

**Authors:** Rui Chen, Hao Zhang, Wantao Wu, Shuyu Li, Zeyu Wang, Ziyu Dai, Zaoqu Liu, Jian Zhang, Peng Luo, Zhiwei Xia, Quan Cheng

**Affiliations:** ^1^ Department of Neurosurgery, Affiliated Nanhua Hospital, University of South China, Hengyang, China; ^2^ Department of Neurosurgery, Xiangya Hospital, Central South University, Changsha, China; ^3^ National Clinical Research Center for Geriatric Disorders, Xiangya Hospital, Central South University, Changsha, China; ^4^ Department of Oncology, Xiangya Hospital, Central South University, Changsha, China; ^5^ Department of Thyroid and Breast Surgery, Tongji Hospital, Tongji Medical College of Huazhong University of Science and Technology, Wuhan, China; ^6^ Department of Interventional Radiology, The First Affiliated Hospital of Zhengzhou University, Zhengzhou, China; ^7^ Department of Oncology, Zhujiang Hospital, Southern Medical University, Guangzhou, China; ^8^ Department of Neurology, Hunan Aerospace Hospital, Changsha, China

**Keywords:** antigen presentation machinery, glioma, microenvironment, prognosis, genomic alteration, immunotherapy

In the original article, there was a mistake in **Figure 4G** as published. The title of **Figure 4G** was mistakenly copyedited from the title of **Figure 4F** during the figure preparation. The corrected **Figure 4** appears below.

**Figure 4:**
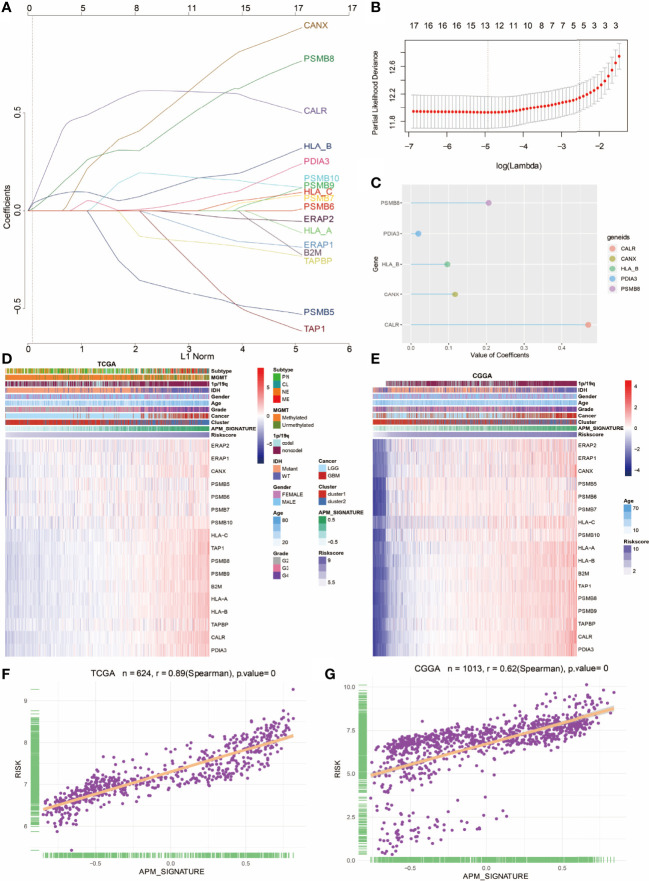
Construction of APM signature gene-based risk score. **(A)** LASSO regression analysis showing the coefficients of APM signature genes. **(B)** Partial likelihood deviance of APM signature genes. **(C)** Bubble plot depicting the APM-derived risk signature genes. **(D)** Heatmap depicting the association between APM signature genes and risk score in the TCGA. **(E)** Heatmap depicting the association between APM signature genes and risk score in the CGGA. **(F)** The correlation between APM signature and risk score in TCGA. **(G)** The correlation between APM signature and risk score in the CGGA.

The authors apologize for this error and state that this does not change the scientific conclusions of the article in any way. The original article has been updated.

In the original article, there was a mistake in **Figure 9E** as published. Images (siRNA-CALR-1 group) were mistakenly chosen from the group named siRNA-NC during the figure preparation. The corrected **Figure 9** appears below.

**Figure 9:**
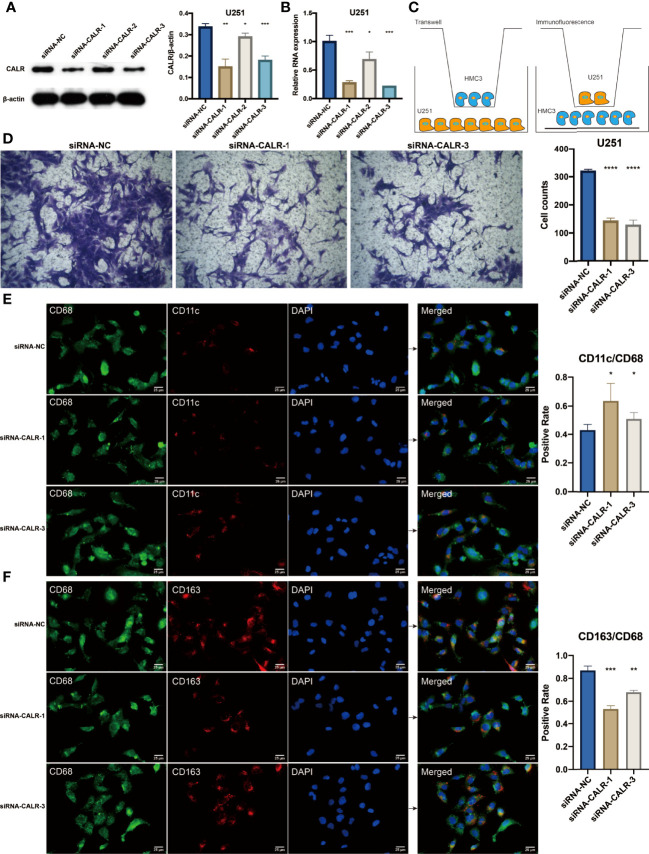
**(A)** Western blotting results for U251 cells treated with siRNAs. Statistical analysis of the western blotting results in different siRNA groups. **(B)** Statistical analysis of the qPCR results in different siRNA groups. **(C)** Diagram for the coculture between HMC3 and U251 cells. **(D)** Transwell assay for the cocultured HMC3 cells. Statistical analysis of the migrated HMC3 cells in different siRNA groups. **(E)** Immunofluorescence staining of CD68 and CD11c in HMC3 cells. **(F)** Immunofluorescence staining of CD68 and CD163 in HMC3 cells. *P < 0.05; **P < 0.01; ***P < 0.001; ****P < 0.0001.

The authors apologize for this error and state that this does not change the scientific conclusions of the article in any way. The original article has been updated.

## Publisher’s Note

All claims expressed in this article are solely those of the authors and do not necessarily represent those of their affiliated organizations, or those of the publisher, the editors and the reviewers. Any product that may be evaluated in this article, or claim that may be made by its manufacturer, is not guaranteed or endorsed by the publisher.

