# Supplementary material for: Antigen Presentation Machinery Signature-Derived CALR Mediates Migration, Polarization of Macrophages in Glioma and Predicts Immunotherapy Response
Source: Front Immunol. 2022 Mar 28;13:833792. doi: 10.3389/fimmu.2022.833792 (PMC8995475; doi:10.3389/fimmu.2022.833792)
Supplement: Supplementary file 1 [file DataSheet_1.docx]

**Figure Legend**


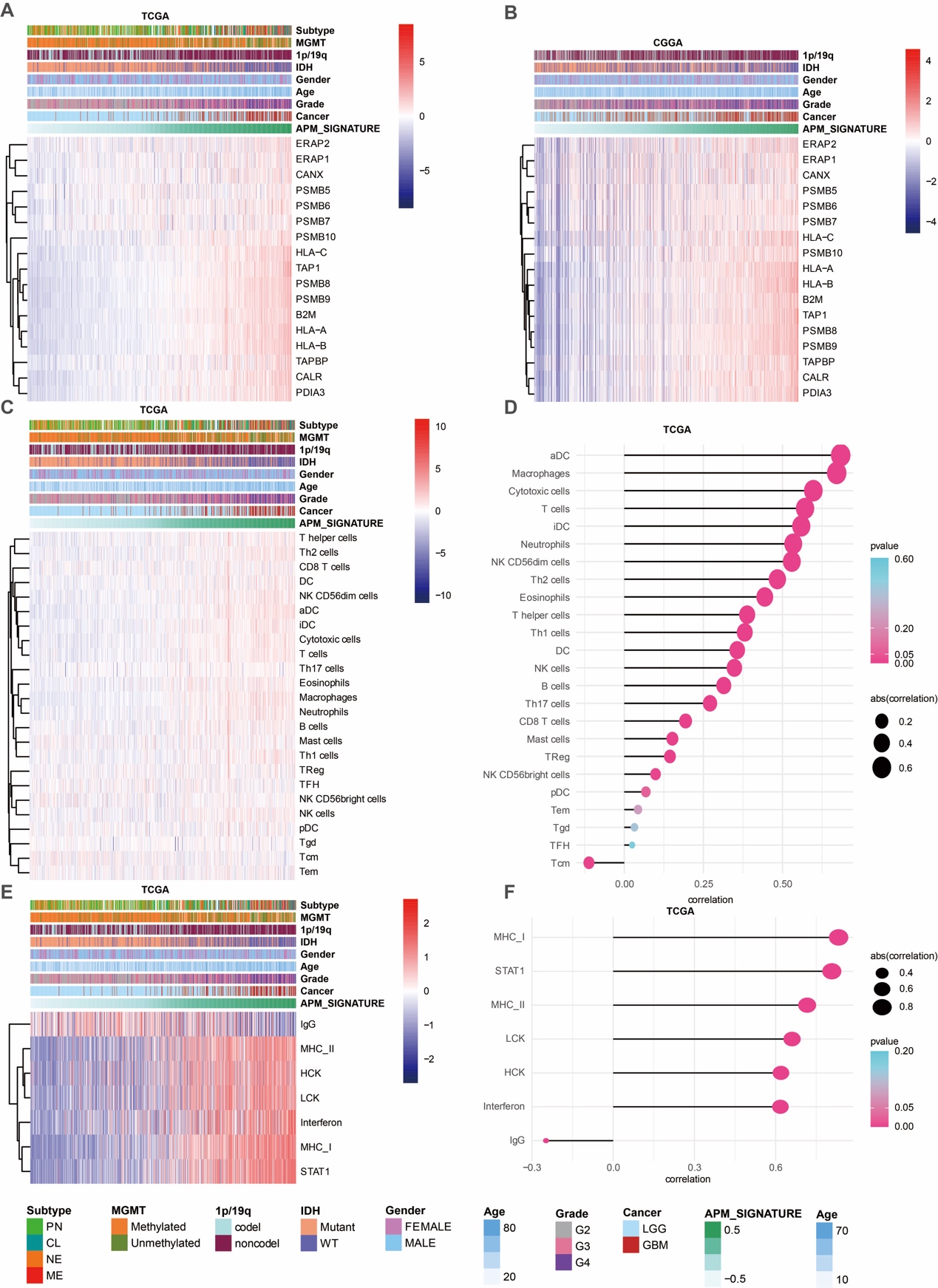


Figure S1. Immunogenic characteristics of APM signature score. A. Heatmap depicting the expression differences of APM signature genes in different levels of APM signature score in TCGA. B. Heatmap depicting the expression differences of APM signature genes in different levels of APM signature score in CGGA. C. Heatmap depicting the abundance of immune infiltrating cells in different levels of APM signature score in TCGA. D. Bubble plot depicting the correlation between APM signature and immune infiltrating cells. E. Heatmap depicting the abundance of inflammatory signature genes in different levels of APM signature score in TCGA. F. Bubble plot depicting the correlation between APM signature and inflammatory signature genes.


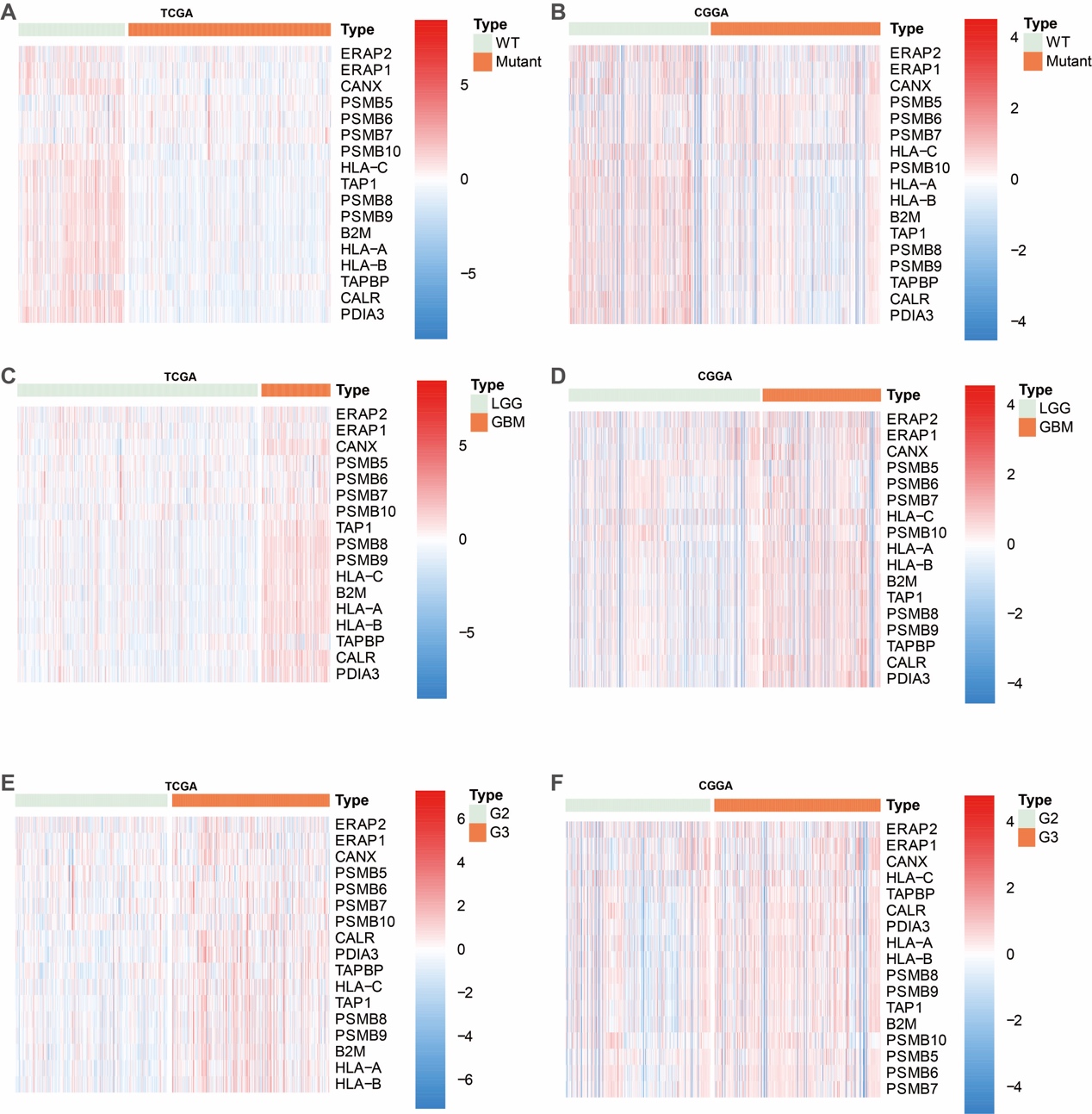


Figure S2. A. Heatmap depicting the expression differences of APM signature genes in IDH mutation and IDH wildtype groups in TCGA. B. Heatmap depicting the expression differences of APM signature genes in IDH mutation and IDH wildtype groups in CGGA. C. Heatmap depicting the expression differences of APM signature genes in LGG samples and GBM samples in TCGA. D. Heatmap depicting the expression differences of APM signature genes in LGG samples and GBM samples in CGGA. E. Heatmap depicting the expression differences of APM signature genes in grade 2 gliomas and grade 3 gliomas in TCGA. F. Heatmap depicting the expression differences of APM signature genes in grade 2 gliomas and grade 3 gliomas in CGGA.


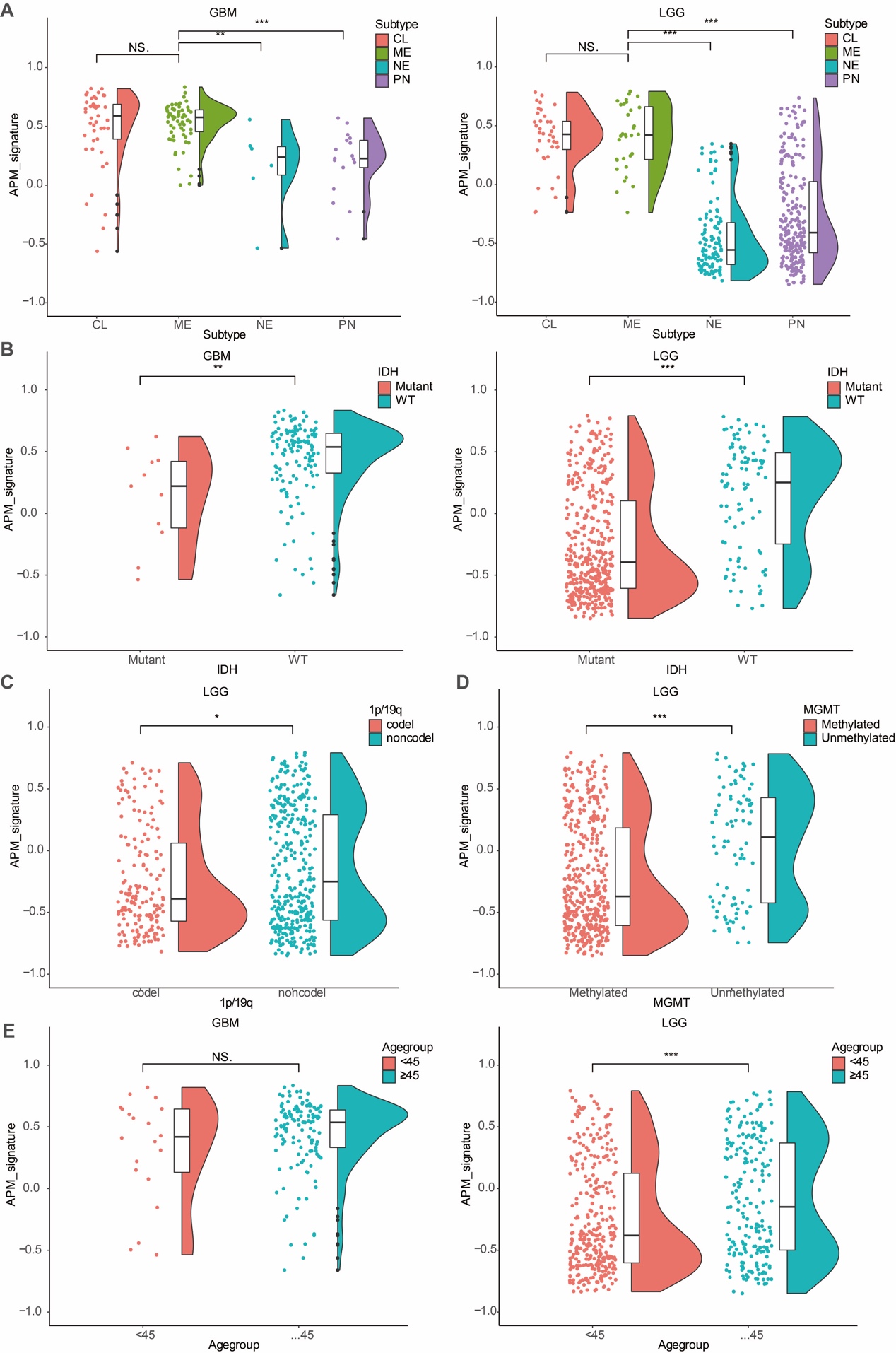


Figure S3. Clinical characteristics and molecular features of APM signature. Raincloud plot depicting the expression differences of APM signature in A. IDH status, B. molecular subtypes, C. 1p19q status, D. MGMT status, E. age groups in LGG and GBM samples, respectively.


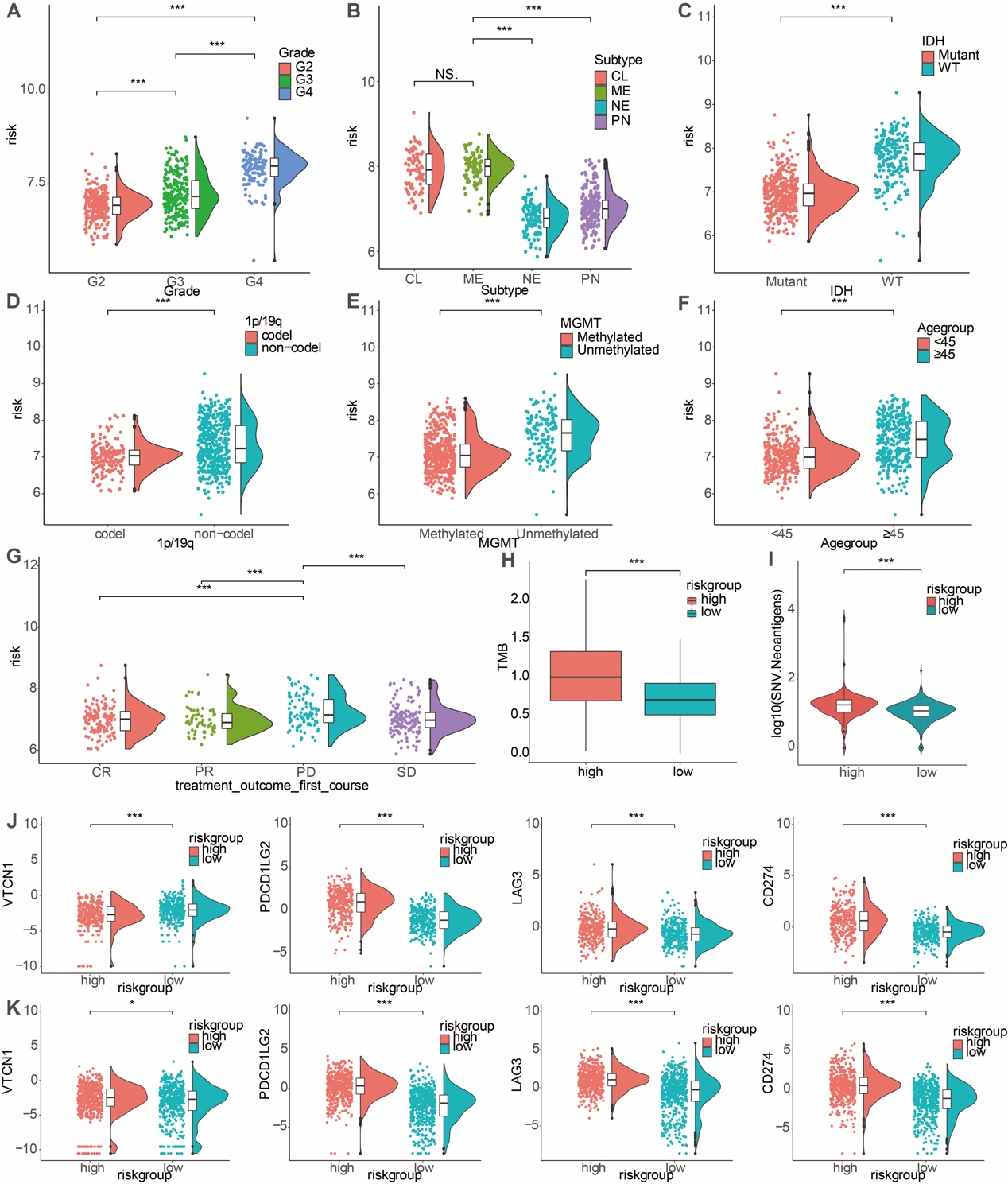


Figure S4. Clinical characteristics and molecular features of risk score. Raincloud plot depicting the expression differences of risk score in A. tumor grade, B. molecular subtypes, C. IDH status, D. 1p19q status, E. MGMT status, F. age groups, and G. treatment outcome. Complete Remission/Response, CR; Partial Remission/Response, PR; Progressive Disease, PD; Stable Disease, SD. H. Different levels of TMB in two risk score groups. I. Different levels of SNV neoantigens in two risk score groups. J. Expression differences of VTCN1, PDCD1LG2, LAG3, and CD274 in two risk score groups in TCGA. K. Expression differences of VTCN1, PDCD1LG2, LAG3, and CD274 in two risk score groups in CGGA.


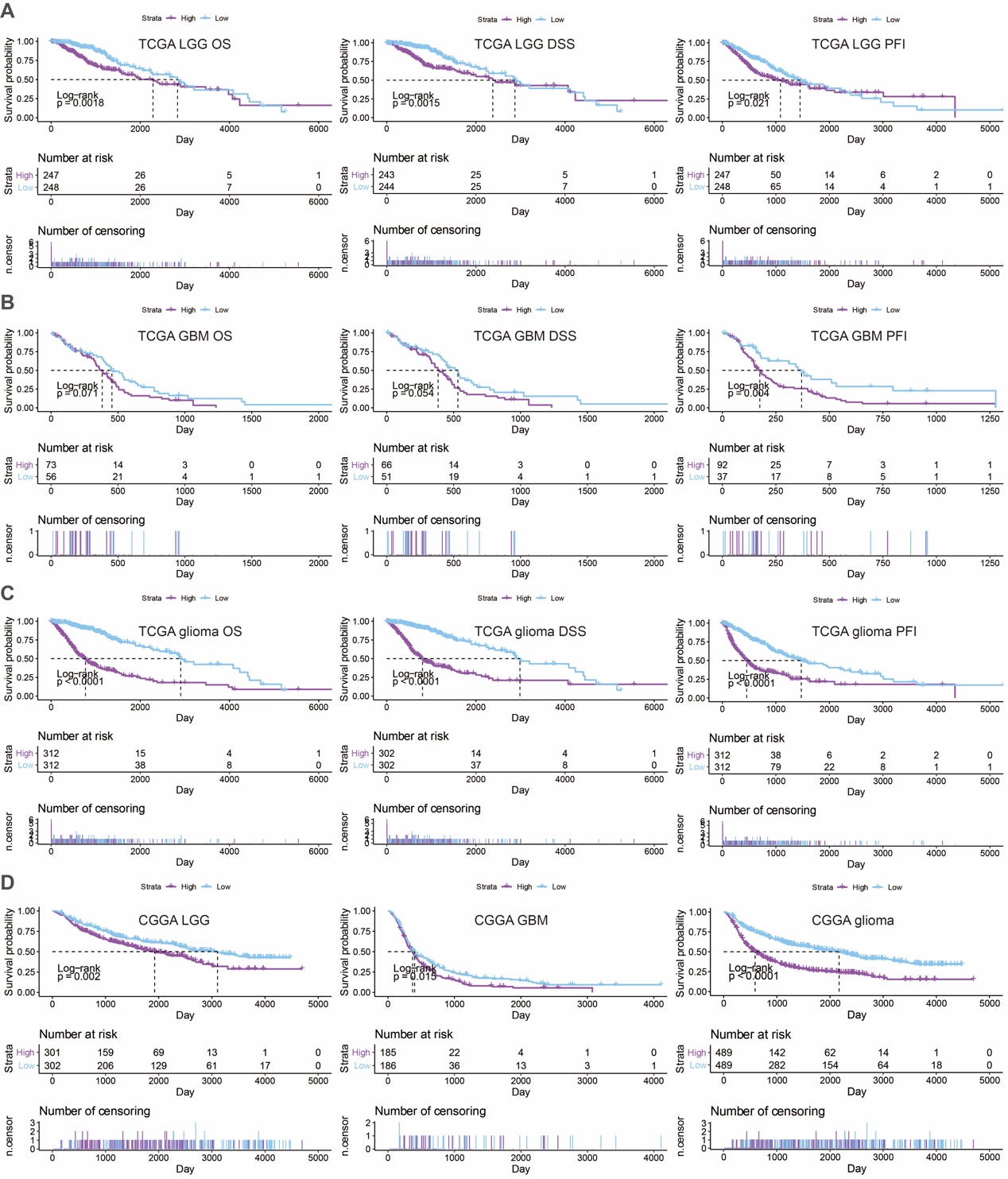


Figure S5. Prognostic value of risk score in A. LGG samples from TCGA, B. GBM samples from TCGA, C. glioma samples from TCGA. D. Kaplan-Meier curves of the two risk score groups regarding OS of glioma samples from CGGA.


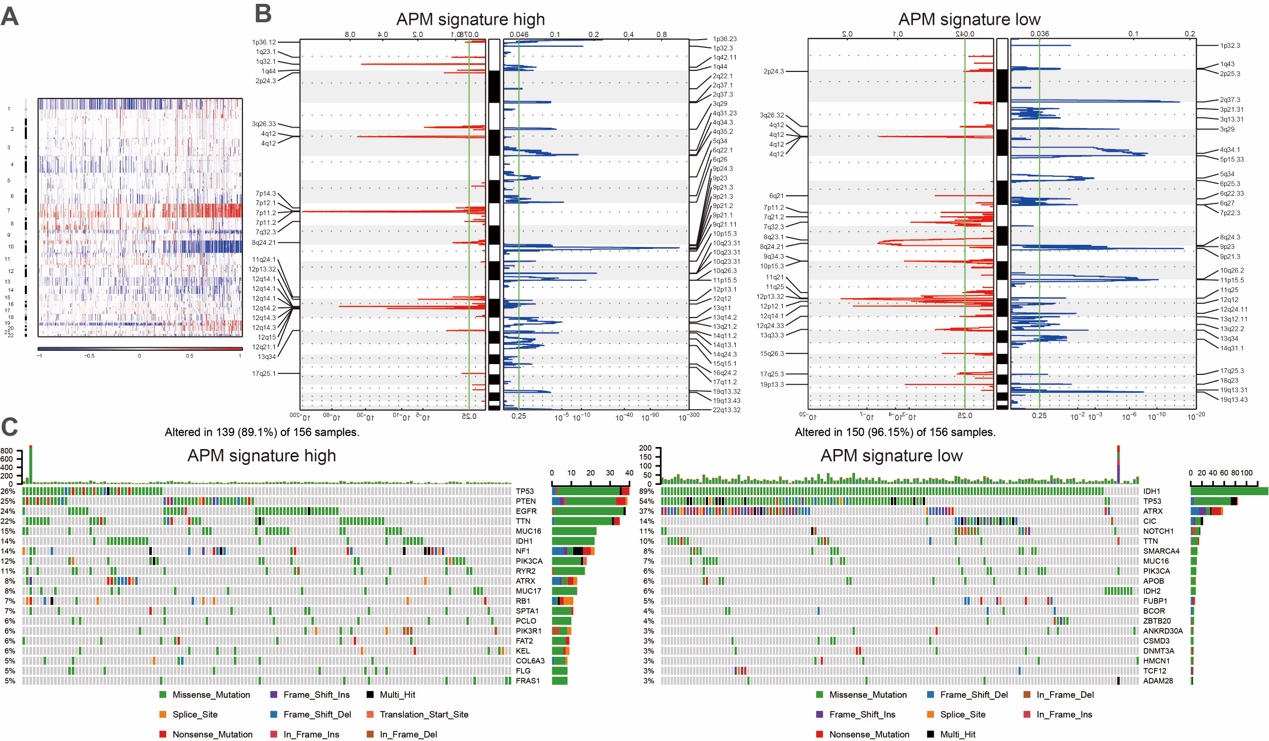


Figure S6. Genomic features of APM signature. A. The overall somatic alteration pattern of glioma. B. Copy number variations in two APM signature groups. C. Genomic alterations in two APM signature groups.


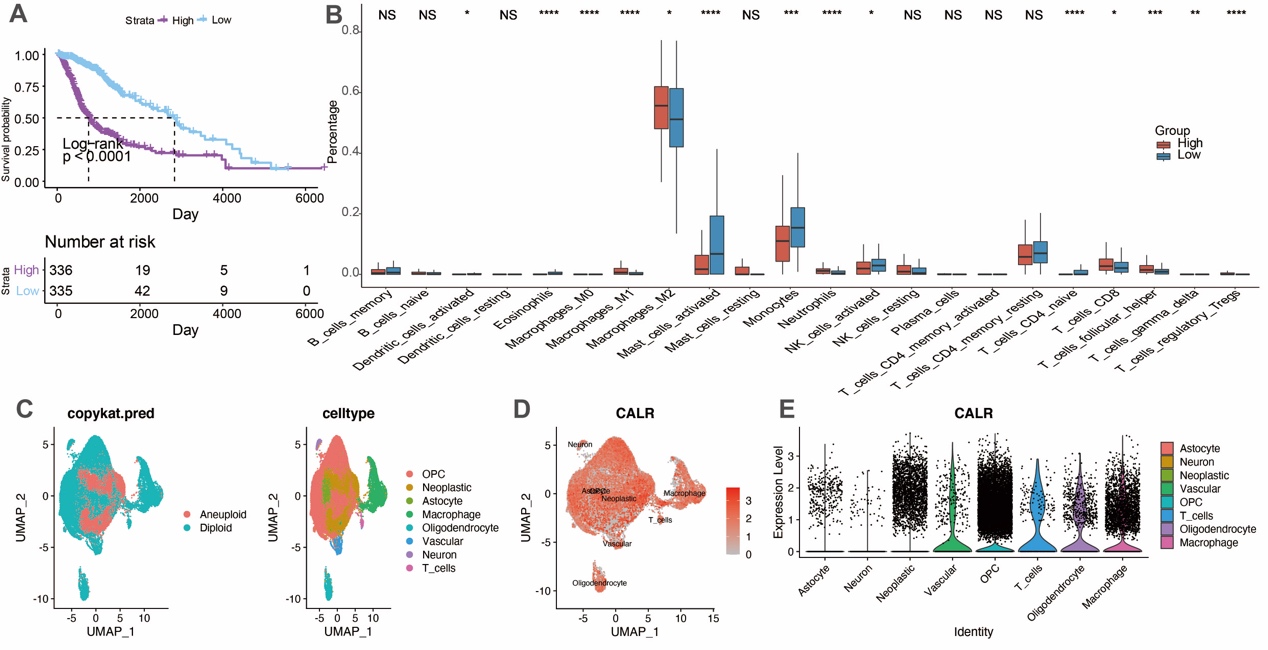


Figure S7. Validation of CALR in tumor microenvironment of glioma. A. Kaplan-Meier curves of the two groups with different expression levels of CALR regarding OS in TCGA. B. Box plot depicting the expression differences of CALR in different immune infiltrating cells based on CIBERSORT algorithm. C. Dimplot for the identified cells in single cell sequencing analysis of GSE138794. D. Featureplot for the expression levels of CALR in identified cells. E. Vlnplot for the expression levels of CALR in identified cells.
